# Association of coral algal symbionts with a diverse viral community responsive to heat shock

**DOI:** 10.1186/s12866-017-1084-5

**Published:** 2017-08-17

**Authors:** Jan D. Brüwer, Shobhit Agrawal, Yi Jin Liew, Manuel Aranda, Christian R. Voolstra

**Affiliations:** 1 Red Sea Research Center, Division of Biological and Environmental Science and Engineering (BESE), King Abdullah University of Science and Technology (KAUST), Thuwal, 23955-6900 Saudi Arabia; 2 Department of Biology and Chemistry, University of Bremen, 28359 Bremen, Germany

**Keywords:** RNA-Seq, Transcriptomics, Virus, *Symbiodinium*, Coral reef, Heat shock, Climate change

## Abstract

**Background:**

Stony corals provide the structural foundation of coral reef ecosystems and are termed holobionts given they engage in symbioses, in particular with photosynthetic dinoflagellates of the genus *Symbiodinium*. Besides *Symbiodinium*, corals also engage with bacteria affecting metabolism, immunity, and resilience of the coral holobiont, but the role of associated viruses is largely unknown. In this regard, the increase of studies using RNA sequencing (RNA-Seq) to assess gene expression provides an opportunity to elucidate viral signatures encompassed within the data via careful delineation of sequence reads and their source of origin.

**Results:**

Here, we re-analyzed an RNA-Seq dataset from a cultured coral symbiont (*Symbiodinium microadriaticum*, Clade A1) across four experimental treatments (control, cold shock, heat shock, dark shock) to characterize associated viral diversity, abundance, and gene expression. Our approach comprised the filtering and removal of host sequence reads, subsequent phylogenetic assignment of sequence reads of putative viral origin, and the assembly and analysis of differentially expressed viral genes. About 15.46% (123 million) of all sequence reads were non-host-related, of which <1% could be classified as archaea, bacteria, or virus. Of these, 18.78% were annotated as virus and comprised a diverse community consistent across experimental treatments. Further, non-host related sequence reads assembled into 56,064 contigs, including 4856 contigs of putative viral origin that featured 43 differentially expressed genes during heat shock. The differentially expressed genes included viral kinases, ubiquitin, and ankyrin repeat proteins (amongst others), which are suggested to help the virus proliferate and inhibit the algal host’s antiviral response.

**Conclusion:**

Our results suggest that a diverse viral community is associated with coral algal endosymbionts of the genus *Symbiodinium*, which prompts further research on their ecological role in coral health and resilience.

**Electronic supplementary material:**

The online version of this article (doi:10.1186/s12866-017-1084-5) contains supplementary material, which is available to authorized users.

## Background

Coral reefs constitute one of the most diverse ecosystems on the planet providing ecological and economical value [1]. These ecosystems are under threat due to the effects of anthropogenic pollution, ocean acidification, and ocean warming, amongst others [1]. Stony corals are the foundation species of reef ecosystems as they provide the structural calcium carbonate framework. They constitute complex assemblages of organisms, termed holobionts, consisting of the animal host organism, photosynthetic dinoflagellates of the genus *Symbiodinium*, bacteria and other microbes, and viruses [2]. In the case of *Symbiodinium* the relationship is a mutualistic symbiosis, as the coral provides a sheltered and light-rich environment, and in return, the algal endosymbionts provide energy to the coral host in the form of photosynthates [3, 4]. The microbial community has been shown to be important for nutrient cycling, in particular nitrogen [5–8], pathogen defense [9], and coral health [10–14]. More recently, studies have started investigating the diversity and function of viruses of the coral holobiont [15–19].

Studies that aimed at characterizing viruses associated with the coral holobiont found a diverse viral community closely associated with coral hosts [16]. The coral surface mucus layer is of particular interest, given that the mucus layer serves as the outer barrier of the coral to the surrounding seawater [20, 21]. Indeed, viral abundances in the mucus layer have been reported to be greater than those in the surrounding seawater by as much as an order of magnitude [22]. Although the exact roles of viruses in the coral holobiont function remain unclear at present, research suggests that viruses might be associated with coral mortality and reef ecosystem decline [16]. For instance, viruses have been proposed to play a role in some coral diseases [19, 23, 24]. Viruses were also proposed to be involved to some degree in coral bleaching, based on a general increase in viral abundance in bleaching corals such as *Diploria strigosa*, *Porites compressa*, *Acropora aspera*, and *Acropora millepora* [15, 17, 25]. Further support comes from a recent study by Levin et al. [26]. In this study, viruses associated with *Symbiodinium* showed differential expression under temperature stress, in particular an increase of nucleocytoplasmic large DNA virus transcripts. However, further research is warranted to unequivocally establish a potential role of viruses in coral disease and coral bleaching.

The study by Levin et al. [26] was interesting in another regard as it assessed viral gene expression by using RNA-Seq data derived from *Symbiodinium* cultures, originally produced to assess algal gene expression [27]. This shows that data from next-generation sequencing studies can be used to investigate gene expression across the range of organisms captured (i.e., organisms associated with the target organism that are sequenced as ‘by-catch’), but only few studies have done so [28–30]. Spurred by the insight and feasibility of these recent studies and approaches, here we re-analyzed a previously published RNA-Seq dataset of cultured *Symbiodinium microadriaticum* (Clade A1) across four experimental conditions for the presence and diversity of viruses and viral gene expression [31]. Our approach included removal of algal host sequence data and phylogenetic annotation of assigned virus sequences as well as assembly and gene expression profiling of putative viral genes, providing a snapshot of virus diversity and viral gene expression. The applied procedure should be amenable to query other RNA-Seq datasets, in particular those obtained from coral holobionts, to extend the insights obtained here.

## Methods

### Algal culturing and experimental treatment

Culturing methods used to generate this dataset are described in detail in Liew et al. [31]. Briefly, individual colonies of *S. microadriaticum* (strain CCMP2467, Clade A1, from Bigelow National Center for Marine Algae and Microbiota) were selected from antibiotic-treated (kanamycin 50 μg/ml, ampicillin 100 μg/ml, and streptomycin 50 μg/ml) f/2-agar plates, and transferred into 200 ml of f/2 media without antibiotics for 2 weeks in non-treated Nunclon Δ TripleFlasks (Thermo Scientific, Waltham, MA). Cultures were exposed to a light intensity of 80 μmol photons m^−2^ s^−1^ on a 12 h:12 h day:night cycle (daytime was set from 6 am to 6 pm). All cultures were sampled at the same time of the day (noon). Cultures were incubated in triplicates under three coral bleaching-relevant stressors alongside a control treatment [31]. Experimental treatments were: cold shock (“16C”: 16 °C for 4 h prior to sampling), heat shock (“36C”: 36 °C for 4 h prior to sampling), and dark shock (“DS”: no light for 6 h prior to sampling). The control treatment (“Noon”) was sampled at 12 pm in the midst of the day cycle.

### RNA extraction and sequencing

For RNA extractions, about 3 × 10^7^ algal cells (200 ml of 1–2 × 10^5^ cells/ml) were pelleted in 50 ml Falcon tubes at 500 *g*, decanted, washed, and re-pelleted at 1000 *g*. The pellets were drip-dried and subsequently snap-frozen with liquid nitrogen for subsequent processing. Total RNA was extracted from the cell pellets via grinding in liquid nitrogen. To aid grinding, 200–300 μl of 0.5 mm glass beads (BioSpec Products, Bartlesville, OK) were added into the mortar. Subsequently, the RNeasy Plant Mini Kit (Qiagen, Hilden, Germany) was used according to manufacturer’s instructions to extract RNA. The quality of RNA was assessed using a Bioanalyzer 2100 (Agilent, Santa Clara, CA) prior to sequencing library creation. Strand-specific RNA-Seq libraries were generated from oligodT-selected total RNA using the NEBNext Ultra Directional RNA Prep Kit (New England Biolabs, Ipswich, MA) according to manufacturer’s instructions. A total of 397 million paired-end read pairs (read length: 101 bp, library insert size: 180 bp) were sequenced and retrieved from the NCBI Short Read Archive (SRA) under BioProject accession PRJNA315758 [31].

### Sequence data filtering

The software trimmomatic [32] was used for quality control and read trimming (settings: headcrop:6; lading:30; trailing:30; slidingwindow:4:30, minlen:35), while fastq-mcf [33] was utilized to remove Illumina adapters (minimum remaining length l = 50, quality threshold q = 20, minimum quality mean qual_mean = 20), retaining 609 of the 795 million sequence reads (397 million paired-end read pairs) (Additional file 1: Figure S1). The BBSplit script from BBMap-35.85 [34] was used to remove sequencing library spiked-in PhiX174 Illumina control sequences. The BBSplit script was also used to remove sequence reads that mapped to the genome of *Symbiodinium microadriaticum* [35, 36]. This yielded 123 million sequence reads (53 million read pairs and 17 million single reads, respectively) that were used for all subsequent analyses (Additional file 1: Figure S1).

### Viral diversity and community composition

Filtered paired-end read pairs (see above) were classified with the software CLARK [37] (Version 1.2.3; default settings; databases: bacteria and viruses; NCBI RefSeq release 75, March 15, 2016). Read pairs from each sample (*n* = 12, i.e. 3 replicates over 4 experimental conditions) were annotated to the highest (taxon) level for classification of sequences as bacteria, archaea, and viruses, and counts for each taxon were summed up for a given treatment and over all treatments. Taxa for which the sum of sequence counts across all samples was <12 (i.e., average count in each replicate <1) were removed from subsequent analyses. Sequence counts of remaining taxa were then utilized to assess changes in viral communities across different experimental treatments. Sequence counts were normalized using the cumulative-sum scaling (CSS) method implemented in the R Bioconductor package metagenomeSeq (v 1.12.1) [38–40], and a zero-inflated log-normal model (*fitFeatureModel* in metagenomeSeq) was calculated. LIMMA empirical Bayes method [41] as implemented in metagenomeSeq was used to test for statistically significant differences for each pairwise comparison between the control (“Noon”) and remaining treatment conditions (cold shock “16C”, heat shock “36C”, dark shock “DS”) at an FDR-corrected cutoff of ≤ 0.1.

### Viral gene assembly, annotation, and gene expression

Besides taxonomic assignment, retained sequence reads (see above) were utilized for a de novo assembly with rnaSPAdes-0.1.1 (default settings) [42]. Assembled contigs (> 200 bp) were annotated using Prokka [43] (settings: -- metagenome, −-norrna --notrna --kingdom Viruses, remaining settings: default; databases: see below), based on predicted coding sequences (CDS) inferred from Prodigal [44] through BLAST+ [45] searches against the viral RefSeq (Release 75, March 07, 2016) [46], the Tara Ocean Virome (TOV) database [47], and a quality-controlled set of viral core genes from UniProt (settings --kingdom viruses, provided by Prokka, release May 2016). Further, HMM profile databases consisting of viral pFAM [48], viral TIGRFAMs [49], vFAMs [50], and Viral OGs of EggNOG 4.5 [51] were queried using hmmscan from the HMMER 3.1 package [52]. For gene expression, retained sequence reads (see above) were mapped to all assembled contigs using Kallisto-0.42.5 [53] (settings: number of bootstrap samples = 10; for single reads: estimated average fragment length = 70, estimated standard deviation of fragment length = 18; remaining settings = default) to retrieve raw counts and Transcripts Per Million (TPM) estimates for each contig across all treatments and replicates. Gene expression was assessed using raw counts, normalized according to the cumulative-sum scaling (CSS) method (see above). A zero-inflated log-normal model was calculated. Differentially expressed genes between the control (“noon”) and remaining treatment conditions (cold shock “16C”, heat shock “36C”, and dark shock “DS”) were determined using the LIMMA Empirical Bayes method [41] as implemented in metagenomeSeq at an FDR ≤ 0.1. Following this procedure, only contigs containing at least one annotated viral gene (i.e., CDS) were considered in the viral gene expression analysis. For graphical visualization, TPM values of significantly differentially expressed virus genes were imported into the Multi Experiment Viewer (MeV) [54] and normalized by expression of genes across conditions (normalized expression = [(TPM) - MeanTPM(across conditions)]/[Standard deviationTPM(across conditions)]) for generation of an expression graph with the R package ggplot2 [55].

## Results

### Viral community composition

A total of 795 million sequence reads (i.e., 397 million paired-end read pairs) across 4 treatments (control, cold shock, heat shock, dark shock) with 3 replicates each (i.e., total of 12 samples), were sequenced. Of these, 123 million sequence reads were retained after quality control and removal of sequence reads that aligned to the genomes of *Microvirus* PhiX174 and *S. microadriaticum* (Table 1, Additional file 1: Figure S1). To assess viral community composition, 263,878 read pairs could be annotated to archaea, bacteria, and viruses (Table 1, Additional file 1: Figure S1). Viruses comprised 18.78%, bacteria 80.45%, and archaea 0.77% of these annotated read pairs (Table 1). Hence, archaea were rare and comprised a minor fraction of the microbial community, whereas bacteria made up the majority of annotated read pairs. In total 144 distinct viral taxa from 41 families (excluding unassigned and unclassified viruses) were identified across all samples (Additional file 2: Table S1).

**Table 1:** Sequence data overview and read-based taxonomic classification

Cond.	Rep.	Raw reads	Retained reads	Classified read pairs (total)	Classified read pairs (virus)	Classified read pairs (bacteria)	Classified read pairs (archaea)
Control	R1	51,620,924	7,594,944	10,067	1128	8821	118
	R2	119,963,940	22,602,385	48,850	8385	40,163	299
	R3	59,952,956	7,914,680	8514	691	7669	154
16C	R1	70,030,018	11,990,261	22,822	1867	12,827	138
	R2	52,277,676	8,772,154	20,585	3031	46,512	282
	R3	51,391,660	7,063,844	9506	680	12,976	176
36C	R1	37,937,408	6,074,958	39,082	18,890	20,790	165
	R2	59,809,100	7,266,991	8646	1444	17,398	156
	R3	70,688,052	9,223,296	10,480	1026	8693	133
DS	R1	55,769,740	8,276,145	14,525	1560	20,071	121
	R2	104,424,430	18,366,941	56,341	9547	7046	156
	R3	61,080,270	7,776,573	14,460	1308	9322	132
	Total	794,946,174	122,923,172	263,878	49,557	212,288	2030
				Percent	18.78	80.45	0.77

Numbers of raw and retained (i.e., after quality filtering, PhiX and algal symbiont removal) sequence reads, as well as number of taxonomically classified read pairs are provided. Retained sequence reads were used for taxonomic and expression analysis

**Fig. 1:**
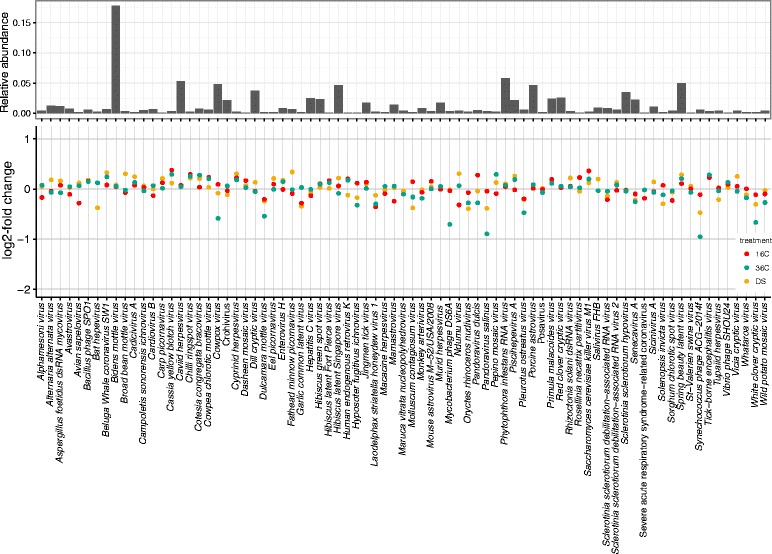
Viral community associated with cultured *Symbiodinium microadriatcum*. Only viral taxa present with ≥ 12 sequence counts (i.e., average count in each replicate ≥ 1) are displayed. Upper portion depicts relative abundance of viral taxa in the control treatment (noon). Lower portion depicts log2-fold changes of taxa-based viral abundance for three shock treatments (cold shock “16C”, heat shock “36C”, and dark shock “DS”) in comparison to control condition

The most abundant viruses were members of the *Potyviridae* family (19.70% ± 1.21%) with a single species (*Bidens mottle virus*) comprising 19.10% ± 1.16% of all annotated read pairs under consideration (Fig. 1, Additional file 2: Table S1). The next five most abundant families were *Picornaviridae* (8.38% ± 0.57%), *Herpesviridae* (8.03% ± 0.51%), *Bromoviridae* (7.73% ± 0.51%), *Astroviridae* (7.27% ± 0.47%), and *Partitiviridae* (7.02% ± 0.41%) (Fig. 1, Additional file 2: Table S1). The viral community was dominated by ssRNA(+) viruses (62.54% ± 4.08%), while dsRNA viruses (22.49% ± 1.36%) and dsDNA viruses (14.88% ± 0.95%) were also present. RNA retroviruses (ssRNA(rt)) and ssDNA viruses were extremely rare (< 0.01% each) and none of ssRNA(−) and dsDNA(rt) were identified. Although some differences between control and treatment conditions could be observed, the abundance of viral species was overall similar and not significantly different (Fig. 1). We found the largest difference in the heat shock treatment (in comparison to the control), with the greatest abundance differences by *Synechococcus phage* ACG-2014f (log2-fold change −0.95), *Pandoravirus salinus* (log2-fold change −0.89), *Mycobacterium phage* DS6A (log2-fold change −0.70), *White clover cryptic virus* (log2-fold change −0.67), *cowpox virus* (log2-fold change −0.59), and *Dulcamara mottle virus* (log2-fold change −0.54) (Fig. 1, Additional file 2: Table S1). Interestingly, all of these viruses became less abundant in the heat shock treatment (Fig. 1, Additional file 2: Table S1).

### Viral gene assembly and expression

To evaluate viral gene expression, 123 million retained sequence reads were assembled into 56,064 contigs (N50 = 1462 bp; > 1000 bp = 15,844 contigs) (Additional file 1: Figure S1). Of these, 5925 contigs were < 200 bp and were not considered for annotation. Of the remaining 50,139 contigs, we predicted 35,271 coding sequences (‘hypothetical proteins’), the majority of which (30,206) did not retrieve an annotation and were not considered for subsequent analyses. Viral gene annotations could be obtained for 4856 contigs. Of these, 2075 contigs were annotated with the viral RefSeq database, 2455 contigs with the Tara Ocean Virome (TOV) database, and 326 contigs with the vFam database.

**Fig. 2:**
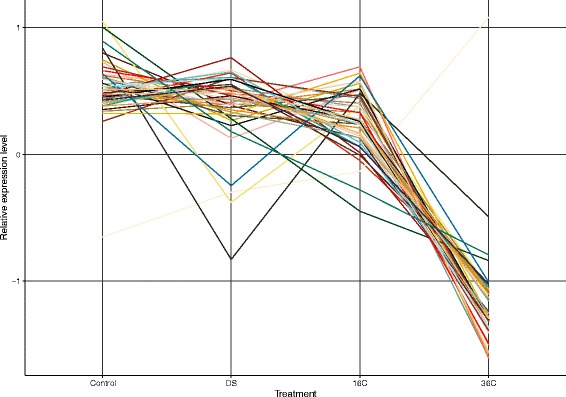
Gene graph of the 43 differentially expressed viral genes in cultures of *S. microadriaticum* across four experimental treatments (control “Noon”, dark shock “DS”, cold shock “16C”, heat shock “36C”). Only genes in the heat shock treatment displayed significant differential expression and were downregulated in comparison to the control (with the exception of one upregulated gene). Displayed are normalized TPM values over replicated treatments. Each gene is represented by a distinct color

To assess viral gene expression, all sequence reads were mapped against the 56,064 contigs and read counts and TPM values were determined (Additional file 1: Figure S1, Additional file 3: Table S2). We found no significant differences in gene expression between control and either cold shock or dark shock treatments for any of the 4856 contigs of putative viral origin. Conversely, for the heat shock treatment, we identified 43 contigs with viral genes that were differentially expressed (Fig. 2), of which 22 received a functional gene annotation (Table 2). Most of these were serine/threonine kinases, ankyrin repeat proteins, and ubiquitins. Similar to the results of viral abundance differences, these genes were downregulated in the heat shock treatment (in comparison to the control) with log2-fold changes ranging from −0.9 to −3.4, with the exception of an ubiquitin E3 ligase ICP0 that was upregulated in the heat shock treatment (log2-fold change 0.8) (Fig. 2, Table 2).

**Table 2:** Set of 22 functionally annotated differentially expressed viral genes in *S. microadriaticum* cultures under heat shock (4 h at 36 °C, FDR < 0.1) and associated log2-fold changes

Putative Viral Gene annotation	log2-fold change
IAP-1 [*Choristoneura occidentalis alphabaculovirus*]	−3.40
ORF MSV197 tryptophan repeat gene family protein [*Melanoplus sanguinipes entomopoxvirus*]	−2.75
ABC transporter [*Bacillus phage* SPBc2]	−2.60
cathepsin [*Adoxophyes orana nucleopolyhedrovirus*]	−2.50
putative serine/threonine-protein kinase [*Acanthamoeba polyphaga mimivirus*]	−2.14
replicase polyprotein 1ab [*White bream virus*]	−2.04
cys/met metabolism plpdependent enzyme [*Pandoravirus inopinatum*]	−2.02
hypothetical protein ATCV1_Z609R [*Acanthocystis turfacea Chlorella virus* 1]	−1.90
putative UDP-galactopyranose mutase [*Sinorhizobium phage* phiM9]	−1.80
ORF58 [*Xestia c-nigrum* granulovirus]	−1.76
tail fiber protein [*Shigella phage* 75/02 Stx]	−1.74
tail fiber protein [*Staphylococcus phage* MCE-2014]	−1.64
mg314 gene product [*Megavirus chiliensis*]	−1.46
ankyrin repeat protein [*Penguinpox virus*]	−1.37
carboxylesterase [*Pandoravirus inopinatum*]	−1.37
cathepsin-like cysteine proteinase [*Spodoptera litura nucleopolyhedrovirus*]	−1.35
glycosyltransferase [*Pithovirus sibericum*]	−1.32
vFam_117 ankyrin repeat [*Pandoravirus salinus*]	−1.11
vFam_117 ankyrin repeat [*Pandoravirus salinus*]	−0.95
predicted protein kinase EsV-1-111 [*Ectocarpus siliculosus virus* 1]	−0.92
putative ankyrin repeat protein [*Acanthamoeba polyphaga mimivirus*]	−0.86
ubiquitin E3 ligase ICP0 [*Felid herpesvirus* 1]	0.83

## Discussion

The importance of bacterial and viral communities to host organisms is being increasingly recognized, but resources for the identification and characterization of viruses are still scarce, making it challenging to identify viruses using a bioinformatics approach [29, 47]. The NCBI RefSeq database, for instance, holds 5539 viral species, while the number of bacterial species is 40,984 (NCBI RefSeq Release 75). Despite these limitations, there is an increase in both the amount and accessibility of genomic and transcriptomic data available for viruses (e.g. NCBI RefSeq, vFam, Tara Ocean Virome, and others). In this regard, two recently published studies focused on identifying new viruses from metagenomic data substantially increased sequence data available for viruses [56, 57]. Although the technical advances of next-generation sequencing provide the basis to study ‘holotranscriptomes’ or ‘hologenomes’ (i.e., the transcriptome or genome of entire metaorganisms), comparatively few studies have focused on or incorporated viruses [28–30, 58].

To complement recent efforts, here we employed a bioinformatics approach to characterize viral community composition and gene expression from an existing RNA-Seq dataset of the coral symbiont *Symbiodinium microadriaticum* [31]. We relied on the circumstance that high throughput sequencing commonly produces data for not only the target organism, but other associated organisms (e.g., microbes and viruses) as well. Importantly, our intention was to annotate viral sequence reads affiliated with the ‘metaorganism’ *Symbiodinium*. Hence, we considered bacteriophages of metaorganism-associated bacteria a specific component of this. Notably, algal cultures were treated with prokaryote-specific antibiotics (kanamycin, streptomycin, and ampicillin) prior to experimental treatments. Thus, we anticipated an overall reduced bacterial contribution as well as a shifted community composition due to variations in susceptibility to the antibiotics by distinct bacteria and archaea. This in turn supposedly had effects on the abundance of assessed bacteriophages. Further, RNA preparations were oligodT-selected prior to library preparation. While dsDNA and ssRNA(+) viruses (including *Herpesviridae*, *Mimiviridae*, and *Polydnaviridae*) polyadenylate their mRNAs [59–61], not all viruses harbor polyadenylated mRNAs (e.g., some ssRNA(−) viruses). Hence, viral community composition using this RNA-Seq dataset is biased towards viruses that harbor polyadenylated mRNAs. In this regard, it is interesting to note that some ssRNA(+) genomes are polyadenylated, including viruses of the *Potyviridae* and *Picornaviridae* family, the two most abundant viral families in the here-analyzed data (Additional file 2: Table S1) [62, 63]. Thus, their particular abundance might come from the circumstance that genomic and transcriptomic sequence data for these viruses were captured. Further, retroviruses and other viral DNA integrated into the genome of *S. microadriaticum* were not assessed, due to our filtering procedure that removed sequences with similarity to the algal host genome. Last, it is important to keep in mind that no viral enrichment steps were applied during sample preparation, explaining the low number of sequences that were of putative viral origin. Still, 49,557 read pairs could be assigned to viruses, which allowed us to obtain an insight into the viral community associated with cultured *S. microadriaticum*, albeit being incomplete and likely biased towards viruses with polyadenylated mRNAs or genomes.

The viruses associated with cultures of *S. microadriaticum* constituted a highly diverse community. Community composition was similar across replicates within treatments and across treatments, thus indicating a stable viral community associated with the algal host (Fig. 1, Additional file 2: Table S1). In total, 41 different viral families were classified, yet only 7 of these families accounted for more than 5% of the community each, highlighting that the viral community was composed of a few abundant and many rare viruses. While previous studies elucidating viruses associated with *Symbiodinium* cultures also detected multiple viral taxa, they found a lower diversity (between 7 to 11 viral families), albeit on the same order of magnitude [26, 29, 30]. The most abundant virus in the here-assessed dataset was the *Bidens mottle virus* of the family *Potyviridae*. Viruses of the family *Potyviridae* are known to infect many different plants, especially economically important species [64–66]. To our knowledge, this is the first report of *Potyviridae* in association with *Symbiodinium*. Other highly abundant families included the *Picornaviridae*, *Herpesviridae*, *Bromoviridae*, *Astroviridae*, and *Partitiviridae* (in decreasing order of abundance). Previous studies investigating viruses associated with *Symbiodinium* found plant-infecting viruses of the family *Phycodnaviridae* [18, 26, 29, 30, 67], which were also present in our study, but at very low abundance.

Interestingly, two different pandoraviruses (*Pandoravirus salinus* and *Pandoravirus dulcis*) were classified. Pandoraviruses are among the largest known viruses, with a particle size > 0.7 μm and a genome size of 1.9 to 2.5 Mb (exceeding some eukaryotes) [68, 69]. Given that the majority of studies use size-based filtration, a filtration-free approach is arguably more likely to detect giant viruses, as suggested previously [26, 30], although Weynberg et al. [67] detected giant viruses of the family *Mimiviridae*, despite applying a size-based filtration.

*Herpesviridae* were the third most abundant virus family in the *Symbiodinium* cultures. Herpes-like viral structures have been described previously in healthy, bleached, and diseased corals [15, 17–19]. However, further analysis only revealed similarities in a few coding regions, suggesting a distinct relationship to known Herpesviruses [16, 25]. Interestingly, a study by Lawrence et al. [70] found viral structures of the same shape and size as the herpesvirus-like particles described above in transmission electron micrographs of a range of *Symbiodinium* cultures and in the nucleus of *Symbiodinium* cells. Additionally, recent studies detected viruses of the order *Herpesvirales* associated with *Symbiodinium* type C1 [67], as well as similarities with viruses of the *Herpesviridae* family of *Symbiodinium* type C3, originally isolated from jellyfish [30]. Accordingly, Herpesviruses previously identified in corals [15–19] might in fact be associated with their algal endosymbionts.

At large, we found stable gene expression of the viral community, which is in agreement with the results of the viral community composition and which parallels the overall stable gene expression of the *Symbiodinium* host [31]. We expected to see increased viral activity in the heat shock treatment, given that a temperature increase might trigger the lytic cycle [24, 71], but we only found few differentially expressed genes. At the same time, we only detected differential gene expression in the short-term heat shock experiment over four hours, and changes might become more apparent in experiments with longer treatment times. Correspondingly, our results suggest that the application of a short-term treatment (over hours), such as heat (“36C”), cold (“16C”), or dark (“DS”) shock, affect viral gene expression to a comparably small extent. Nevertheless, some viral genes were downregulated in the heat shock treatment (with the exception of one upregulated gene), while the cold and dark shock treatments did not show any effect on viral gene expression. Given that the amount of differentially expressed genes was < 1% of all genes assayed, we do not attribute this to a general shutdown of the host transcriptional machinery under heat shock, but rather as a viral response to the heat shock.

The differentially expressed viral genes included serine/threonine kinases, ankyrins, ABC transporters, and ubiquitins, of which ankyrins and ubiquitins have been reported as upregulated after UV exposure in C3 *Symbiodinium* cultures [30]. Further, kinases, ankyrins, and ABC transportes have been described in the virome associated with cultured *Symbiodinium* recently [67]. In the context of viral infections, kinases are known to impact and regulate the autophagy of cells [72–74], which in turn constitutes part of an innate and adaptive immune response of organisms [72, 75]. Two serine/threonine kinases (Us1 and Us3) of Herpesviruses were shown to inhibit interferons of the respective host, thus hinder the autophagy and immunity of the host cell [74, 76, 77]. Further, (de-)ubiquitination enzymes have a key role in antiviral immunity [78, 79]. Further, the replication of many viruses depends on the hydrolytic activity of the ubiquitin-proteasome system in order to make amino acids available for viral protein synthesis [80–82]. ABC transporters, however, are not known to affect viral reproduction, but might present an alternative mechanism to counteract the host’s innate immunity [83, 84]. Ankyrin repeat proteins are one of the most abundant and ubiquitous proteins in all kingdoms of life and harbor structurally and regulatory important roles, yet they are rare in viruses [81, 85]. Ankyrin repeat proteins have been described as being part of signaling networks during and after viral infections [85, 86] as well as to counteract the innate immunity of host cells by preventing apoptosis of the host and manipulating the host’s ubiquitination system [78, 81, 87]. Taken together, the cold shock and dark shock treatments did not elicit significant differences regarding viral gene expression, while the heat shock treatment resulted in the downregulation of some viral genes (< 1% of all genes assayed). Most of these genes seem to play a role in inhibiting the host’s antiviral response, but it remains to be determined whether this is due to the host or the viruses being affected by the heat shock. Of note, we did not observe significant viral abundance changes between treatments and individual viral taxa seem to be similar abundant across all treatments (Fig. 1, Additional file 2: Table S1). As such, the downregulation of genes could not be associated with the decreased abundance of certain viruses.

Coral bleaching (i.e., the loss of *Symbiodinium*) affects coral reefs on a global scale, and bleaching events are primarily due to an increase in seawater temperature [88]. Nevertheless, the mechanisms are not yet fully understood [1, 7, 89–92]. Heat stress has been previously implicated in the onset of viral outbreaks and it has been suggested that heat-induced increases in viral activity may be associated with some aspects of coral bleaching [15–18, 22, 25, 93]. Along these lines, Wilson et al. [94] showed temperature induction of viruses in *Symbiodinium*, and Levin et al. [26] found differential viral gene expression in *Symbiodinium* populations that coincided with their thermal tolerance. Conversely, Weynberg et al. [67] did not find differences in viral abundance in cultured *Symbiodinium* type C1 after exposure to elevated temperature. Here we found a relatively stable viral community, but could detect differences in the viral gene expression during a short-term heat shock treatment. Taken together, while a potential role of viruses in coral bleaching is compelling, conclusive data supporting this is unavailable at the moment. Future targeted efforts should help clarify the role of viruses associated with the coral holobiont and any presumptive impacts on coral health and resilience.

## Conclusions

Despite the putative importance of viruses, comparatively few studies describe the viral diversity associated with eukaryotic hosts. Host-targeted RNA-Seq studies provide an opportunity to examine associated viral signatures. In this study, we used an oligodT-selected transcriptome dataset obtained from antibiotic-treated cultured *Symbiodinium microadriaticum* under three shock treatments and one control treatment to assess viral diversity and gene expression. From these data, we observed a stably associated and diverse viral community that is characterized by few abundant and many rare viral families. The most abundant viral family were the *Potyviridae*, which encompasses many known plant viruses, and has not been described in *Symbiodinium* before. Expression of genes in the viral community was primarily affected by heat shock and included processes targeting host antiviral immunity, but the relevance of this to the coral holobiont remains to be shown. Taken together, we present an approach to assess viral communities and gene expression from existing RNA-Seq data that can be applied to existing high throughput gene expression datasets.

## Additional files

Additional file 1: Figure S1. Analysis pipeline to assess viral diversity and gene expression from RNA-Seq data. After sequence read filtering, the pipeline is split for viral community and differential gene expression examination. Software used depicted in *italics* with specified settings (if other than default). (PDF 436 kb)

Additional file 2: Table S1. Taxonomic diversity of viral classified sequences. Paired reads were classified with CLARK. Shown are CSS-normalized counts over viral taxa for each replicate of four experimental conditions: control, cold shock (“16C”), heat shock (“36C”), and dark shock (“DS”). (XLSX 38 kb)

Additional file 3: Table S2. Viral gene expression in Transcripts Per Million (TPM). TPM values for all contigs with viral genes (*n* = 4856) for each replicate of four experimental conditions: control, cold shock (“16C”), heat shock (“36C”), and dark shock (“DS”). (XLSX 825 kb)

## Abbreviations

RNA-Seq: RNA sequencing
