# Supplementary material for: Association of coral algal symbionts with a diverse viral community responsive to heat shock
Source: BMC Microbiol. 2017 Aug 17;17:174. doi: 10.1186/s12866-017-1084-5 (PMC5561611; doi:10.1186/s12866-017-1084-5)
Supplement: Supplementary file 1 — Analysis pipeline to assess viral diversity and gene expression from RNA-Seq data. After sequence read filtering, the pipeline is split for viral community and differential gene expression examination. Software used depicted in italics with specified settings (if other than default). (PDF 436 kb) [file 12866_2017_1084_MOESM1_ESM.pdf]

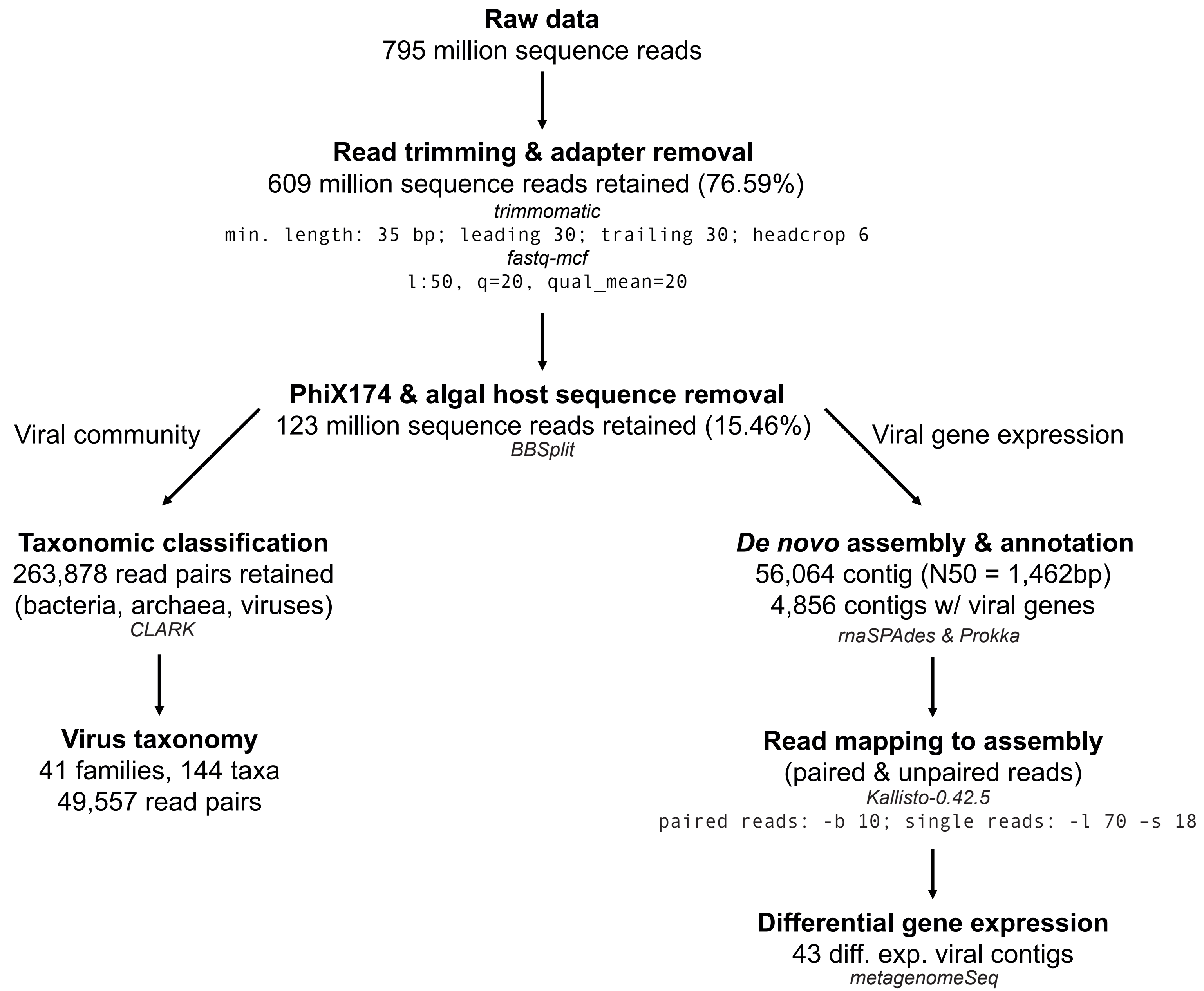
Raw data
795 million sequence reads
Read trimming & adapter removal
609 million sequence reads retained (76.59%)
trimmomatic
min. length: 35 bp; leading 30; trailing 30; headcrop 6
fastq-mcf
l:50, q=20, qual_mean=20
PhiX174 & algal host sequence removal
123 million sequence reads retained (15.46%)
BBSplit
Viral community
Viral gene expression
Taxonomic classification
263,878 read pairs retained
(bacteria, archaea, viruses)
CLARK
Virus taxonomy
41 families, 144 taxa
49,557 read pairs
De novo assembly & annotation
56,064 contig (N50 = 1,462bp)
4,856 contigs w/ viral genes
rnaSPAdes & Prokka
Read mapping to assembly
(paired & unpaired reads)
Kallisto-0.42.5
paired reads: -b 10; single reads: -l 70 -s 18
Differential gene expression
43 diff. exp. viral contigs
metagenomeSeq
